# Proangiogenic properties of complement protein C1q can contribute to endometriosis

**DOI:** 10.3389/fimmu.2024.1405597

**Published:** 2024-06-25

**Authors:** Chiara Agostinis, Miriam Toffoli, Gabriella Zito, Andrea Balduit, Silvia Pegoraro, Mariagiulia Spazzapan, Lorella Pascolo, Federico Romano, Giovanni Di Lorenzo, Alessandro Mangogna, Aurora Santin, Beatrice Spedicati, Erica Valencic, Giorgia Girotto, Giuseppe Ricci, Uday Kishore, Roberta Bulla

**Affiliations:** ^1^ Institute for Maternal and Child Health, IRCCS Burlo Garofolo, Trieste, Italy; ^2^ Department of Medical, Surgical and Health Science, University of Trieste, Trieste, Italy; ^3^ Department of Life Sciences, University of Trieste, Trieste, Italy; ^4^ Department of Veterinary Medicine, United Arab Emirates University, Al Ain, United Arab Emirates; ^5^ Zayed Centre for Health Sciences, United Arab Emirates (UAE) University, Al Ain, United Arab Emirates

**Keywords:** C1q, complement system, endometriosis, angiogenesis, gC1qR, ovary, endothelial cells

## Abstract

Endometriosis (EM) is defined as the engraftment and proliferation of functional endometrial-like tissue outside the uterine cavity, leading to a chronic inflammatory condition. While the precise etiology of EM remains elusive, recent studies have highlighted the crucial involvement of a dysregulated immune system. The complement system is one of the predominantly altered immune pathways in EM. Owing to its involvement in the process of angiogenesis, here, we have examined the possible role of the first recognition molecule of the complement classical pathway, C1q. C1q plays seminal roles in several physiological and pathological processes independent of complement activation, including tumor growth, placentation, wound healing, and angiogenesis. Gene expression analysis using the publicly available data revealed that C1q is expressed at higher levels in EM lesions compared to their healthy counterparts. Immunohistochemical analysis confirmed the presence of C1q protein, being localized around the blood vessels in the EM lesions. CD68^+^ macrophages are the likely producer of C1q in the EM lesions since cultured EM cells did not produce C1q *in vitro*. To explore the underlying reasons for increased C1q expression in EM, we focused on its established pro-angiogenic role. Employing various angiogenesis assays on primary endothelial endometriotic cells, such as migration, proliferation, and tube formation assays, we observed a robust proangiogenic effect induced by C1q on endothelial cells in the context of EM. C1q promoted angiogenesis in endothelial cells isolated from EM lesions (as well as healthy ovary that is also rich in C1q). Interestingly, endothelial cells from EM lesions seem to overexpress the receptor for the globular heads of C1q (gC1qR), a putative C1q receptor. Experiments with siRNA to silence gC1qR resulted in diminished capacity of C1q to perform its angiogenic functions, suggesting that C1q is likely to engage gC1qR in the pathophysiology of EM. gC1qR can be a potential therapeutic target in EM patients that will disrupt C1q-mediated proangiogenic activities in EM.

## Introduction

Endometriosis (EM) is a chronic disease characterized by the presence of functional endometrium outside the uterine cavity (1). It is a very frequent gynecological disorder that affects around 10–15% of reproductive-aged women, with an overall prevalence of about 18% (2, 3). EM is commonly associated with pelvic pain, dyschezia, dyspareunia, dysmenorrhea, and infertility (4). The ectopic tissue forms lesions, characterized by a unique microenvironment involving inflammation and angiogenesis. According to pathophysiology and localization, the endometriotic lesions consisting of endometrial glands and stroma can be divided into three different types: superficial peritoneal EM, ovarian endometrioma (OMA), and deep infiltrating EM (5).

EM often presents symptoms that mimic other conditions, thus, delaying its early diagnosis. The time between symptom onset and EM diagnosis is usually long and the negative impact of this delay (between 6 to 10 years worldwide, irrespective of the healthcare system) can seriously affect patient’s lifestyle and clinical outcomes (6, 7) A careful history of menstrual symptoms and chronic pelvic pain provides a basis for suspecting EM. No screening tools or tests are currently validated for EM prediction.

Treatment for EM vary, based on symptom severity and a desire to conceive. No current treatment permanently cures the disease. Medical therapy is commonly initiated for pain control without surgical confirmation of the disease. Such treatment is intended to reduce pain through various mechanisms, including minimizing inflammation, interrupting or suppressing cyclic ovarian hormone production, inhibiting the action and synthesis of estradiol, and reducing or eliminating menses. Surgical approaches to relieve EM-related pain can be used as the first-line therapy, or initiated after failed medical therapies (8).

Retrograde menstruation is the most widely accepted pathological mechanism of the onset of EM (9). However, not all women affected by retrograde menstruation develop EM, suggesting a multifactorial cause, including defective immune surveillance (10). Several studies have suggested that EM is due to aberrant immune activation (*e.g.*, immune cell recruitment, cell adhesion and exaggerated inflammatory processes), facilitating the implantation and survival of endometriotic lesions (11, 12).

The complement system is a crucial component of the humoral immunity that acts as a bridge between innate and adaptive immunity (13, 14). Complement is involved in host defense against infectious agents and altered self, playing a pivotal role in the activation of inflammatory processes. It has been shown that the complement system is one of the most impaired pathways in EM, although the mechanisms underlying its involvement in the pathogenesis of EM have not been fully elucidated (15).

Beyond the canonical functions carried out by complement, alternative roles of several complement components have emerged, particularly with regard to C1q (16, 17). C1q is the first recognition subcomponent of the complement classical pathway. It is a complex protein composed by 18 chains organized in a tulip bouquet–like structure comprising a triple-helical collagen-like region and C-terminal globular heads (18). An emerging complement activation-independent function of C1q concerns its capability to promote angiogenesis. It has been reported that C1q on its own can act as a proangiogenic factor in placental development, wound healing, post-stroke *restitutio ad integrum*, and tumor progression (19). In animal models, C1q deficiency is correlated with pre-eclampsia-like symptoms, a gestational syndrome characterized by angiogenic dysfunction; thus, C1q seems crucial during normal development of the placental tissue (20). Moreover, Bossi et al. demonstrated a pro-angiogenic role of C1q in the wound healing process, being localized in granulation tissue, independent of complement activation, based on permeability, proliferation, and migration of endothelial cells (ECs) (21). C1q is also involved in the angiogenic process during post-stroke ischemia recovery. On the other hand, few studies demonstrated that C1q could also drive tumour angiogenesis (22, 23).

It is widely recognized that blood supply is essential for the survival of endometriotic implants and further development of EM, since it can provide nutrients and growth factors, and promote recruitment of inflammatory cells to the endometriotic lesions (24). Microvascular ECs are also active participants in inflammatory processes via secretion of pro-inflammatory mediators and modulation of adhesion and migration of leukocytes through the expression of adhesion molecules and chemokines (25), in addition to being crucial players in angiogenic processes. As demonstrated by Agostinis et al., ECs may respond very differently to pro-inflammatory and pro-angiogenic stimuli depending on their tissue of origin (25).

In this study, we aimed to investigate the presence of C1q in endometriotic lesions and its potential proangiogenic role in EM. Thus, we evaluated its effects in classical angiogenic processes, such as cell motility, proliferation, and tube formation in ECs isolated from the EM lesions as compared to ECs isolated from healthy ovaries, the most frequent site of EM engraftment.

## Methods

### Reagents and antibodies

The following antibodies were used: mouse mAb anti-human CD34 (#MA5–15331), Alexa Fluor 647 donkey anti-mouse IgG (H+L) (#A-31571), Alexa Fluor 594 donkey anti-goat IgG (H+L) (#A-11058), and Alexa Fluor 488 donkey anti-rabbit IgG (H+L) (#A-21206) were purchased from ThermoFisher Scientific (Massachusetts, USA); rabbit mAb anti-human CD31/PECAM1 (#ZRB1216) from Sigma Aldrich (Missouri, USA); mouse mAb anti-human vWF (#M0616), rabbit pAb anti-human vWF (#IS527), rabbit mAb anti-human CK8/18 (#M3652), mouse mAb anti-human CD31/PECAM1 (#M0823), mouse anti-human CD68 (#14–0688-80), and goat anti-mouse FITC-conjugated F(ab)’ (#F0479) from Dako (Milan, Italy); mouse mAb anti-human VE-cad was kindly provided by E. Dejana (Mario Negri Institute, Milan, Italy); FITC-conjugated goat anti-rabbit (#11–4839-81) was purchased from Jackson ImmunoResearch (Milan, Italy); mouse anti-human CD31 FITC (#557508) was purchased from BD Bioscience (New Jersey, USA). Other chemicals were purchased from Sigma Aldrich.

### Gene expression profiling analysis

The analysis of C1q gene expression levels via its three chains *(C1QA, C1QB*, and *C1QC*) in control endometrium and different EM lesions (peritoneal, deep, and OMA) was conducted using data obtained from the Gene Expression Omnibus of the National Centre for Biotechnology Information (NCBI) with the accession number GSE141549. Microarray analysis on samples obtained from 43 endometrium biopsies of healthy woman, 101 endometrium biopsies of EM patients and 190 EM lesions, and data normalization, have been described by Gabriel and colleagues (26). Stages were defined following the rASRM classification (27).

### Patient enrollment and specimen collection

Women included in this study were recruited from the Endometriosis Clinic at the I.R.C.C.S. ‘Burlo Garofolo’ Hospital (Trieste, Italy). All patients underwent surgical removal of ovarian endometriotic cysts through laparoscopy and subsequently received histological confirmation of the diagnosis. Patients positive for human immunodeficiency virus-1, hepatitis B virus, hepatitis C virus, or other sexually transmitted diseases, and patients with co-morbidities, including peritoneal neoplasms, teratomas, endometrial polyps or any other proliferative diseases, were excluded from the study. During laparoscopy, endometrial biopsies were obtained using VABRA aspirator. The severity of EM was assessed using the revised American Society for Reproductive Medicine (rASRM) classification. All participants signed an informed consent form, following the approval of the Ethics Committee of the Friuli-Venezia Giulia region (Italy) (Protocol N 0023422/P/GEN/ARCS 2021). Upon enrollment, detailed information regarding medical history (e.g., age of menarche, EM diagnosis, family history of EM, number of pregnancies, and infertility diagnosis) and EM symptoms (e.g., ovulation, pre-menstrual and post-menstrual pain, dysmenorrhea, dyspareunia, dyschezia, and dysuria) were collected.

Tissue samples collected from EM lesions or endometrial biopsies were fixed in 10% buffered formalin and embedded in paraffin for immunohistochemical (IHC) analysis. A section of the lesion (about 1 cm^2^) was minced and preserved in Trizol (Invitrogen) for total RNA isolation, and a section was sliced for cell isolation. Prior to surgery, whole blood sample was collected in an EDTA-containing vacutainer tube for a group of patients and immediately processed for immunophenotyping.

Healthy ovarian samples were obtained from patients undergoing ovariectomy/annessiectomy during sex reassignment surgery, or patients suffering from gynecological disorders unrelated to ovaries (e.g., leiomyomas). Patients were enrolled at the Institute for Maternal and Child Health, IRCCS Burlo Garofolo, Trieste, Italy. All participants signed an informed consent form, following the approval of the Regional Ethical Committee of FVG (CEUR), Udine, Italy (Prot. 0010143/P/GEN/ARCS 2019).

### Immunophenotyping by flow cytometry

Prior to surgery for ovarian endometriotic cyst removal, whole blood samples were collected in an EDTA-containing vacutainer and processed within 2h. Antibodies against human-CD45 FITC, human-CD3-PerCP-Cy5.5, and human-CD56-PE (1:50) were added to 100 μl of sample and incubated for 30 min at room temperature (RT) in the dark. Red blood cells were then lysed with High-Yield Lyse Fixative-Free Lysing Solution (Life Technologies) for 15 min. Labeled samples were acquired using Attune NxT Flow Cytometer (ThermoFisher) equipped with a 488 nm laser, and analyzed with Attune Cytometric Software v5.3.0. Gating strategy used to identify and characterize CD56^+^CD3^-^ Natural Killer (NK) cells is shown in **Supplementary Figure 1**.

### Cell isolation and culture

Human umbilical vein ECs (HUVECs) were isolated and cultured, as described by Tedesco et al. (28). ECs were isolated from both endometriotic ovarian cysts (EECs) and healthy ovarian biopsy (OVECs), following the procedure described by Agostinis et al. (29). The protocol was slightly modified for the isolation of OVECs. Briefly, the tissue was minced and digested with 0.5% trypsin (Sigma-Aldrich) (0.1% for OVECs) and 50 μg/ml DNase I (Roche, Milano, Italy) overnight at 4°C, and then with collagenase type 1 (3 mg/ml) (Worthington Biochemical Corporation, DBA, Milano, Italy) for 30 min - (20 min for OVECs) at 37°C. After assessing cell viability with Trypan blue, ECs were positively selected with a mix of Dynabeads^®^ CD31 (4x10^8^ beads/mL, Invitrogen; Thermo Fisher Scientific-11155D) and Dynabeads™ M-450 (Life Technologies, Milan, Italy) coated with Ulex europaeus 1 lectin (Sigma-Aldrich), and then seeded on a fibronectin-gelatin coated T12.5 cm^2^ flask. Cells were maintained in Human Endothelial Serum Free Medium (HESFM, Gibco, Carlsbad, CA) supplemented with 1% Penicillin-Streptomycin (PS), 20 ng/mL basic Fibroblast Growth Factor (bFGF), 10 ng/mL Epidermal Growth Factor (EGF), 10% v/v fetal bovine serum (FBS, Life Technologies), and 10% v/v normal human serum (NHS; Sigma-Aldrich). For OVECs, hydrocortisone (1:100; Sigma Aldrich) was added. For the endometriotic lesions, the CD31^-^ epithelial/stromal cells (EMCs) were also kept and seeded on a gelatin-coated flask with HESFM + 10% FBS. Cells were maintained at 37°C in a humified atmosphere in a 5% *v/v* CO_2_ incubator, and the culture medium was changed every 2–3 days depending on the growth of the cells.

### Flow cytometry analysis and immunofluorescence for primary cell characterization

ECs (5x10^5^) were fixed in 3% v/v paraformaldehyde (PFA) in dark for 15 min. Next, primary antibodies: anti-human CD31/PECAM1-FITC (1:25), anti-human vWF (1:50), and anti-human CK8/18 (1:50), were incubated for 45 min at 4°C, diluted in Saponin A (Farmitalia Carlo Erba, Milan, Italy) when permeabilization was needed, or in PBS + 1% (*w/v*) BSA for cell membrane staining. Incubation with FITC-conjugated anti-mouse and anti-rabbit secondary antibodies (1:300 in Saponin A) was performed for 30 min on ice in the dark. Cells were resuspended and fixed in 1% PFA. Fluorescence was acquired using Attune NxT Flow Cytometer (ThermoFisher) equipped with a 488 nm laser, and analyzed with the Attune Cytometric Software v5.3.0.

For immunofluorescence characterization, cells were seeded on round glass coverslips of 13 mm diameter and allowed to reach 70% of confluency. Cells were then fixed with 3% PFA for 15 min at RT in the dark and washed twice with PBS+ 0.1% Tween 20. For blocking, permeabilization and quenching simultaneously, cells were incubated with a buffer containing PBS + 1% *w/v* BSA + 0.1% Triton X-100 + 50 mM glycine for 30 min at RT. After washing primary antibodies were added in PBS + 2% *w/v* BSA for 1h at RT. Cells were washed again twice and incubated with Cy3-conjugated secondary antibodies (1:300) for 30 min in the dark. During the last 5 min of incubation, DAPI was added (1:1000). Coverslips were then washed twice and mounted on glass slides with a drop of Fluorescence Mounting Medium. Images were acquired with Leica DM3000 microscope and Leica DFC320 camera.

### Immunohistochemistry

Tissue samples were fixed in 10% buffered formalin and paraffin embedded. 4–5µm tissue sections were deparaffinized with xylene and rehydrated with decreasing percentage of ethanol (100%, 95%, 70%) and H_2_O. Antigen retrieval was performed for 20 min at 98.5°C in Tris-HCl/EDTA buffer, pH 9.0, for C1q, and in Citrate buffer, pH 6.0, for C4d and CD133 staining. Neutralization of the endogenous peroxidases was performed by adding H_2_O_2_ for 5 min; the blocking of nonspecific binding was achieved via incubation with PBS+2% *w/v* bovine serum albumin (BSA) for 30 min. Sections were incubated overnight at 4°C with primary antibodies, rabbit anti-human C1q (1:500), rabbit anti-human C4d (1:100), or rabbit anti-human CD133 (1:50) diluted in PBS, followed by incubation with anti-rabbit horseradish peroxidase (HRP)-conjugate (1:500) for 30 min at RT. Staining was performed by 3-amino-9-ethylcarbazole (AEC) chromogen substrate (Vector Laboratories). Sections were counterstained with Mayer haematoxylin (DiaPath) and examined under a Leica DM 2000 optical microscope. Images were acquired using Leica DFC 7000 T digital camera (Leica Microsystems, Wetzlar, Germany).

### Double-staining immunofluorescence of paraffined section

Paraffined tissue sections of endometriotic ovarian lesions were deparaffinized with xylene and rehydrated with decreasing ethanol percentage (100%, 95%, 70%) and H_2_O. Antigen retrieval was performed in Tris-HCl/EDTA buffer, pH 9.0, for 20 min at 98.5°C. Slides were incubated with PBS + 1% *w/v* BSA + 0.01% Triton X-100 for 1h to prevent nonspecific binding. Primary antibodies diluted in the blocking buffer were added in the following combination: rabbit anti-human C1q (1:300) and mouse anti-human CD68 (1:100), rabbit anti-human C1q and mouse anti-human vWF (1:50), and rabbit anti-human C1q and mouse anti-human CD34 (1:50). After overnight incubation at 4°C, the anti-rabbit-Cy3 (1:300) and anti-mouse-Alexa Fluor 488 (1:750) secondary antibodies were added for 2h at RT. After 10 min of incubation with DAPI (1:500), slides were mounted using a Fluorescence Mounting Medium (Dako, Golstrup, Denmark), and images were acquired with a Leica DM 2000 (Leica, Wetzlar, Germany) fluorescence microscope using a Leica DFC 7000 T digital camera.

### Gene expression analysis

Tissue samples from endometriotic lesions were minced and lysed using Trizol reagents (1 mL for 50–100 mg of tissue). Total RNA extraction was carried out using the PureLink™ RNA Mini Kit (Invitrogen, ThermoFisher). For isolated primary cells, RNA extraction was performed by lysing the cells with RNA Lysis Buffer and using the Total RNA Purification kit (Norgen Biotek Corp., Thorold, Canada), following the manufacturer’s protocol. Isolated RNA was then quantified using NanoDrop™ 2000/2000c spectrophotometer (ThermoFischer Scientific, Massachusetts, USA) and reverse transcribed in to cDNA using SensiFAST™ cDNA Synthesis kit (Meridian Life Science, Memphis, USA). For RT-qPCR, Power SYBR™ Green Master Mix (Applied Biosystems, Life Technology, USA) was used and the reaction was performed using Corbett Rotor-Gene™ 6000 (Qiagen, Hilden, Germany). Expression levels of the human C1qA, C1qB, and C1qC genes were assessed through a comparative quantification analysis, considering reaction efficiency and normalization against the housekeeping gene, glyceraldehyde-3-phosphate dehydrogenase (GAPDH) (**Table 1**).

**Table 1:** List of primers used for Real-Time quantitative PCR.

Gene	Melting Temperature (C°)	Forward sequence Reverse sequence	Accession number
*C1QA*	60	TGGAGTTGACAACAGGAGGC CGATATGGCCAGCACACAGA	NM_001347465.2
*C1QB*	60	ACCCCAGGGATAAAAGGAGAG GGCAGAGAAGGCGATTTTCTG	NM_001371184.3
*C1QC*	60	AGGATGGGTACGACGGACTG CTTCTGCCCTTTGGGTCCTC	NM_001347619.2
*GAPDH*	60	CCAGGTGGTCTCCTCTGACTT GTTGCTGTAGCCAAATTCGTT	NM_001357943.2

### Surface biotinylation assay

OVECs and EECs (1x10^6^/well) were seeded on to a 6-well plate. The following day, cells were washed with ice-cold PBS + 1 mM MgCl_2_ + 0.1 mM CaCl_2_ and then incubated with EZ-LinkTM- Sulfo-NHS-Biotin (1 mg/mL) for 30 min on ice on an orbital shaker. To remove the excess of biotin, quenching was performed with 0.1 M glycine for 5 min at RT with shaking. Cells were collected with a scraper and washed thrice with ice-cold PBS. Cell pellets were resuspended into RIPA buffer (1% NP-40, 150 mM NaCl, 10 mM Tris-HCl pH. 7.4, 0.1% protease inhibitors), kept on ice for 20 min, and then centrifuged at 14,000 x g for 10 min at 4°C. From each lysate, an input (1:10) was taken and resuspended into 2X Laemmli buffer. The remaining lysates were incubated with High Capacity Streptavidin Agarose Resin (50% slurry, 0.02% sodium azide; Thermo Scientific) on a rotary shaker for 2h at 4°C. Streptavidin resin was washed three times in RIPA buffer, followed by centrifugation at 5,000 x g for 5 seconds. After the last washing step, the supernatant was carefully removed and 20 μL of 2 x Laemmli buffer was added to the resin to elute the biotinylated cell surface proteins. Samples were either stored at -80°C, or used immediately for Western Blot analysis.

### Western blot analysis

Cell lysate proteins were separated on a 10% SDS-PAGE under reducing conditions and transferred to a nitrocellulose membrane using the semi-dry transfer apparatus Trans-Blot Turbo System (BIO-RAD). After 1h of incubation with 5% skimmed milk in PBS + 0.1% Tween 20, the membrane was probed with anti-gC1qR (2 μg/mL; a kind gift from Prof Berhane Ghebrehiwet, State University New York, Stony Brook, NY, USA) and anti-actin antibodies overnight at 4°C. Membrane was washed three times, every time for 5 minutes, and then incubated with LI-COR IRDye secondary antibodies (1:20,000 dilutions of anti-rabbit IgG IRDye^®^ 800CW and anti-mouse IgG IRDye^®^ 680RD) for 1h at RT. After three washing steps, the fluorescence intensity was measured by the Odyssey^®^ CLx near-infrared scanner (LI‐COR Biosciences, Lincoln, NE, USA). Image acquisition, processing and data analysis were performed using Image Studio 5.2 (LI-COR Biosciences).

### Angiogenesis assays

Migration assay was performed in a trans-well system using EECs, OVECs or HUVECs. Cells were stained with the fluorescent dye FAST DiI™ and seeded in FluoroBlok™ Inserts (1.5x10^5^ cells/insert). The lower chamber of the trans-well was loaded with VEGF (20 ng/mL), as a positive control, or C1q (10 μg/mL). After 18h, the fluorescence was read with INFINITE 200 Fluorescence Plate Reader, applying a bottom reader protocol. The percentage of migration was compared against a calibration curve established with an increasing number of labeled cells.

Wound healing assay with primary ECs was performed after seeding 5x10^4^ cells/well in a 24-well plate. After reaching a confluence of 60–70%, the endothelial monolayer was perturbed at the center of the well with a tip. Then, cells were stimulated with VEGF (20 ng/mL) as a positive control, or C1q (10 μg/mL), and maintained at 37° in a 5% v/v CO_2_ incubator. Images of the wound fields were captured at different time points (0 and 8h) and the percentage of wound closure was compared among the different conditions.

Tube formation assay with primary ECs was performed by seeding 5x10^4^ cells in a CultureSlides onto a Matrigel^®^ drop and stimulated with VEGF (20 ng/mL), as a positive control, or C1q (10 μg/mL). After 18 h, using TiEsseLab BDS 600 microscope, the capillary-like structures formed by ECs in the Matrigel^®^ matrix were counted, comparing the different conditions.

Proliferation assay with primary ECs was performed seeding 7x10^3^ cells/well in a 96-well plate and then stimulating them with VEGF (20 ng/mL), as a positive control, or C1q (10 μg/mL). After 24h, MTS was added to each well and cell proliferation was measured using PowerWave Select X Microplate Reader (450 nm).

### Binding of C1q to endothelial cells

ECs were grown to confluence in 96-well tissue culture plates (Corning) and incubated with C1q (10 μg/mL), for 5, 15, 30, 60, or 120 min at RT. Bound C1q was probed by polyclonal rabbit anti-human C1q (1:300; Dako) and alkaline phosphatase-conjugated secondary antibody (1:20,000; Sigma–Aldrich). The enzymatic reaction was developed using p-nitrophenyl phosphate (Sigma–Aldrich; 1 mg/mL) as a substrate and read kinetically at 405 nm using a Titertek Multiskan ELISA reader (Flow Labs).

### Statistical analysis

Data were analyzed by GraphPad Prism software 5.0 (GraphPad Software Inc., La Jolla, CA, USA). Unpaired two-tailed Mann-Whitney test was used for the analysis of C1q gene expression and for GEP analysis; paired t test was applied for angiogenesis experiments. Results were expressed as mean ± standard error mean (SEM) of three independent experiments performed in duplicate. P-values <0.05 were considered statistically significant.

## Results

### Gene expression analyses reveal higher levels of C1q expression in EM lesions compared to patient-derived uterine endometrium

**Figure 1:**
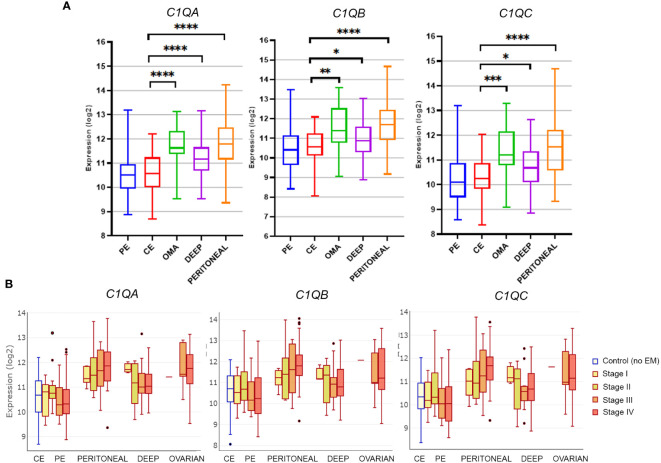
Gene expression analysis of C1q in EM lesions based on EndometDB. **(A)** Histograms representing *C1QA*, *C1QB*, and *C1QC* mRNA expression in control endometrium (CE), patient endometrium (PE), and in different EM lesions (peritoneal; deep; and ovarian, OMA). Gene expression profiling (GEP) analysis, based on data extracted from GEO (GSE141549), revealed a significantly higher expression of all three C1q genes in EM lesions as compared to CE. **(B)** Analysis of C1q gene expression in EM patients clustered into different disease stages (stage I-IV). *p < 0.05, **p < 0.01, ***p < 0.001, ****p < 0.0001 (Mann-Whitney U Test).

To gauge C1q expression in EM, we interrogated the publicly available database EndometDB developed by the Turku University (Finland), which collected gene expression data from 190 EM patients and 43 healthy controls (26). Gene expression profiling (GEP) analysis revealed that all the three genes of C1q (*C1QA*, *C1QB*, and *C1QC*) were up-regulated in all histotypes of EM lesions (peritoneal, deep, and OMA) compared to healthy control endometrium (CE). As shown in **Figure 1A**, significantly lower C1q expression levels were detected in CE or in patient-derived endometrium (PE) as compared to EM lesions, especially when analyzing peritoneal EM and OMA. In these last two histotypes, an association between C1q expression level and disease severity (stage I-IV) was also observed, although it was not statistically significant (**Figure 1B**).

### Demographic data and clinical features and immunophenotype of EM patients

A cohort of EM patients (*n* = 20), aged between 30 and 57 years, was enrolled at the I.R.C.C.S. “Burlo Garofolo” Hospital (Trieste, Italy). The participating patients’ demographic data and clinical features are listed in **Table 2**. Of note, most of the patients (15/20; 75%) were diagnosed with stage III–IV, according to the standard classification of the revised American Society for Reproductive Medicine (rASRM) (27). Furthermore, 60% of the EM patients had full-term pregnancies, while 25% received a diagnosis of infertility. At the time of enrollment, 50% of patients were undergoing hormonal therapies (*i.e*., progestins, combined oestrogen–progestins, or hormone-releasing intrauterine devices). To immunophenotype circulating leukocytes in EM patients, we focused on natural killer (NK) cells. It is widely regarded that the uterine environment is partly regulated by uterine NK (uNK) cells, which are characterized by lower cytotoxicity (CD16^low^, CD56^bright^); they play a key role in establishing a suitable *milieu* for embryo implantation. The uNK cells in the peritoneal fluid as well as uterine endometrium of women affected by EM showed a lower cytotoxic activity. In the EM lesions, it revealed a lower capability to induce apoptosis in endometrial cells (30); this reduction seemed associated with the severity of the disease (31). Based on CD56 expression, NK cells were classified in CD56^++/bright^ and CD56^+/dim^ subsets, since the CD56^++/bright^ CD16^−^ NK subset represents pre-terminally differentiated NK cells, capable of proliferation and cytokine/proangiogenic factor production with low cytotoxic properties (32). Interestingly, a significantly higher percentage of circulating CD56^++/bright^ NK cells was observed in EM patients compared to healthy women (**Supplementary Figure 1**). Conversely, no significant differences in CD45^++^, CD3^+^, or total NK percentage were observed between EM patients and healthy controls (**Supplementary Table 1**).

**Table 2:** Demographic characteristics of the enrolled EM patients.

EM Patients (*n* = 20)		
Age, years (SD)	32.5 (7.3)	
Age at menarche, years (SD)	12.5 (1.6)	
Body-mass index, kg/m^2^ (SD)	22.1 (2.8)	
Fertility status	*n*	%
Pregnancy	12	60%
Infertility	5	25%
n.a.	3	15%
rASRM classification	*n*	%
Stage I	2	10%
Stage II	3	15%
Stage III-IV	15	75%
Adenomyosis	*n*	%
Yes	4	20%
No	16	80%
Ongoing medical therapy	*n*	%
Yes	10	50%
No	10	50%

Stages were defined following the revised American Society for Reproductive Medicine (rASRM) classification (27). n, number; n.a., not available; SD, standard deviation.

### Distribution of C1q in endometriotic lesions, eutopic endometrium, and healthy ovary

**Figure 2:**
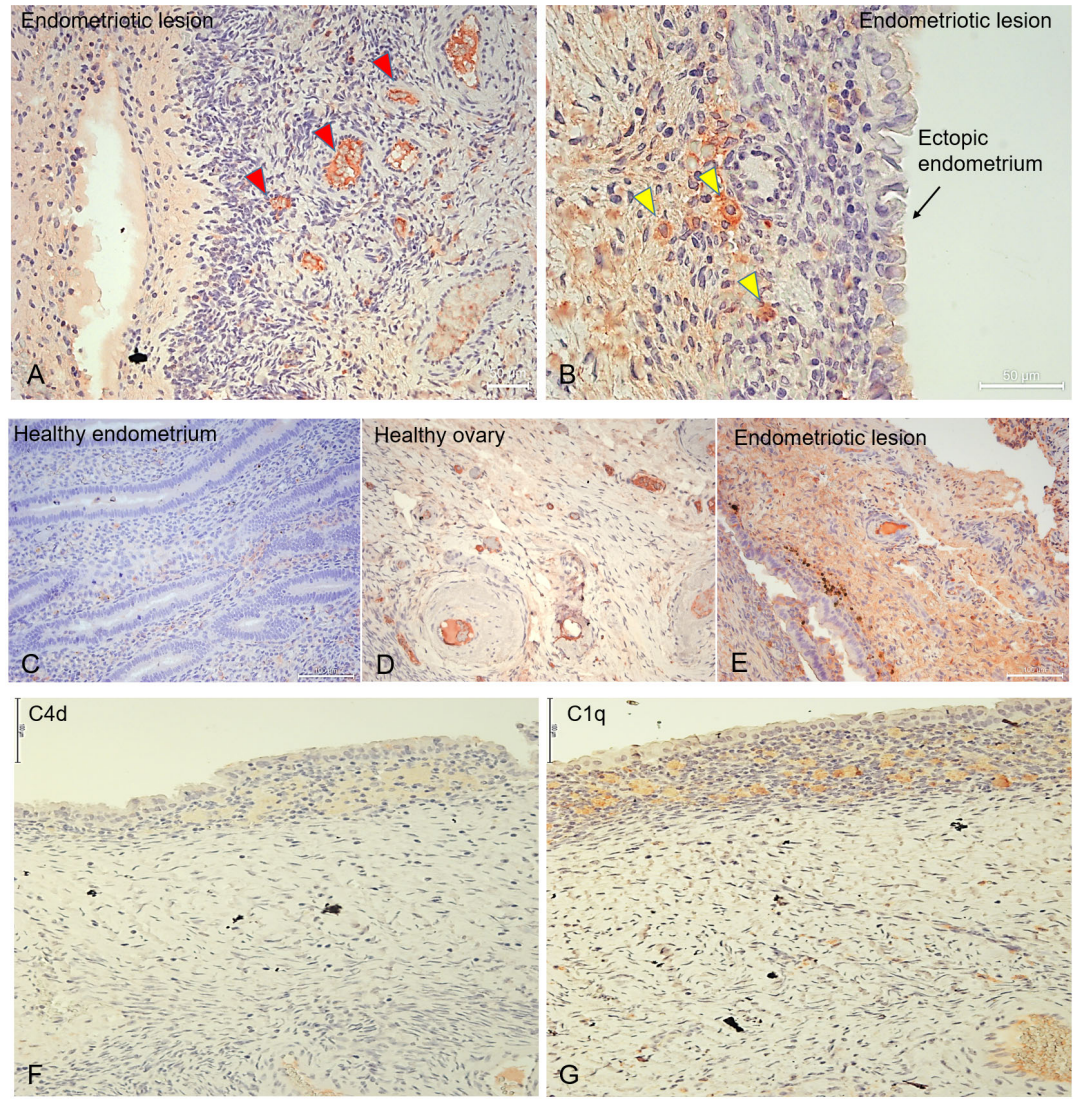
C1q is abundantly present in endometriotic lesions and in healthy ovary. Representative microphotographs showing the presence of C1q in ovarian endometriotic lesions **(A, B, E)**, patients’ eutopic endometrium **(C)**, and healthy ovary **(D)**. AEC (red) chromogen was used to visualize the binding of rabbit anti-human C1q antibody. Red arrows indicate vessels **(A)**, while yellow arrows indicate isolated cells scattered in EM stroma which resulted due to positive staining for C1q **(B)**. **(F, G)** Representative microphotographs showing the presence of C4d **(F)** or C1q **(G)** in serial sections of endometriotic lesions. C1q is present in the endometriotic lesion; however, the classical pathway is feebly activated. AEC (red) chromogen was used to visualize the binding of secondary antibodies. Nuclei were counterstained in blue with Harris Hematoxylin. Magnification, 10x **(A, C-E)**; 20x **(B)**. Scale bars, 50 µm **(A, B)**; 100 µm **(C-G)**.

We investigated the local distribution of C1q in ectopic and eutopic endometrium via immunohistochemistry (IHC) using EM tissue samples. C1q displayed an intense positivity around the vessels in endometriotic lesions (**Figure 2A**; red arrows). C1q staining was particularly evident in some isolated cells scattered in the stroma (**Figure 2B**; yellow arrows). However, eutopic endometrium of the EM patients (**Figure 2C**) (and from healthy women) (**Supplementary Figure 2**) was almost negative for C1q staining. We also analyzed C1q expression in the ovary, which is the most frequent site of EM engraftment. Surprisingly, we observed C1q expression in healthy ovary tissue (**Figure 2D**), even though the staining was considerably weaker compared to endometriotic lesions (**Figure 2E**). To understand whether C1q presence could be associated with the classical pathway activation, we investigated also C4d expression. We noticed a weak positivity for C4d; however, its intensity was not comparable to C1q staining (**Figures 2F, G**).

**Figure 3:**
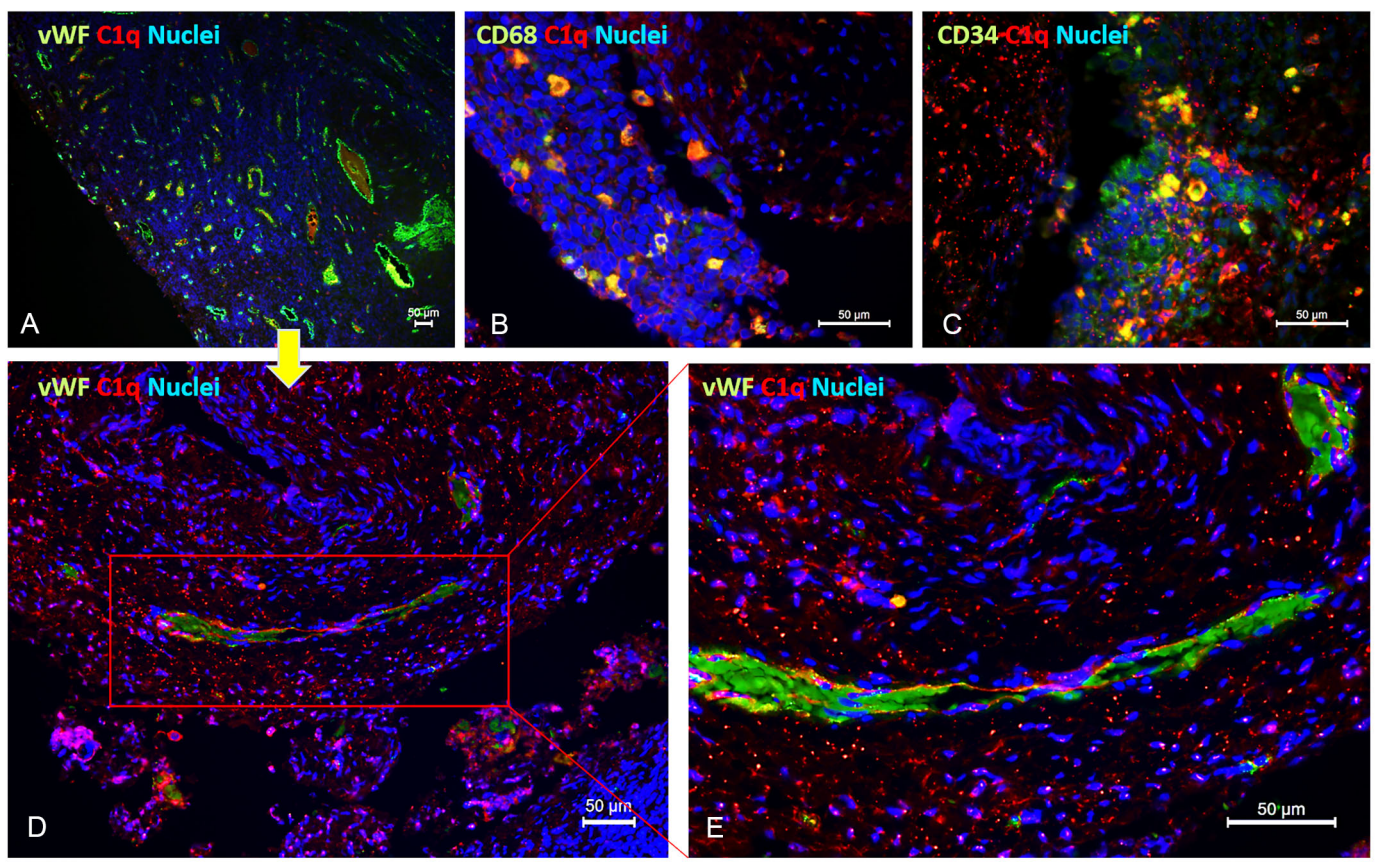
Double immunofluorescence microscopy for C1q in EM lesions. Representative images showing double staining for C1q (red) and vWF **(A, D, E)**, CD68 **(B)**, or CD34 **(C)** (green) in endometriotic lesions. After deparaffinization, tissue sections were incubated overnight with anti-human C1q and anti-human vWF, CD68, or CD34 primary antibodies, followed by incubation with anti-rabbit Cy3 and anti-mouse Alexa Fluor™ 488 secondary antibodies. Cell nuclei were stained with DAPI. Scale bars, 50 µm.

Unlike most complement components that are synthesized by hepatocytes, C1q is known to be primarily produced by macrophages (and adherent monocytes); however, it seems that under certain circumstances, ECs are capable of expressing C1q or binding it (19). To discern the cells capable of synthesizing C1q within the microenvironment of endometriotic lesions, we conducted double immunofluorescence (IF) analysis for C1q and von Willebrand Factor (vWF) as an EC marker (**Figure 3A**), CD68 as a macrophage marker (**Figure 3B**), or CD34 as a marker of newly formed vessels or endothelial progenitor cells (EPCs) (**Figure 3C**). As shown in **Figure 3**, several CD68^+^ and CD34^+^ cells exhibited a positive staining for C1q. Furthermore, co-labelling with vWF revealed that the C1q staining was associated with mature endometriotic endothelium (**Figures 3D, E**). To exclude the expression of C1q by CD34^+^ EPCs, we analyzed also the presence of CD133^+^ cells in the EM tissue. As shown in **Supplementary Figure 3**, we failed to detect the presence of CD133^+^ cells in EM lesions.

### C1q transcripts are locally expressed in endometriotic lesions but not in isolated primary cells

**Figure 4:**
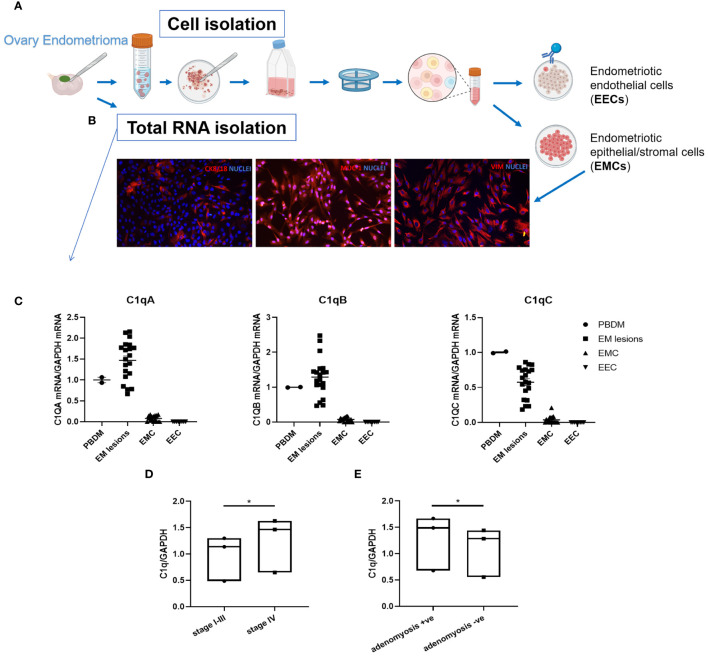
*C1QA*, *C1QB*, and *C1QC* gene expression in endometriotic lesions and primary isolated endometriotic cells. **(A)** Graphical representation of primary EM cell isolation procedure. **(B)** Characterization of endometriotic cells (EMCs) isolated from EM ovary cysts by immunofluorescence. Around 10% of EMCs resulted positively stained for cytokeratin (CK)8/18, and 100% were positive for mucin-1 (MUC-1) and vimentin. Cell nuclei were stained with DAPI. **(C)** After total RNA extraction and retro-transcription, C1q gene expression was analyzed by performing RT-qPCR. Peripheral blood mononuclear cells (PBMCs) were used as positive control. GAPDH was used as the housekeeping gene. Scatter plots were generated with the software GraphPad Prism 8.4.3. **(D, E)** The clustering of EM patients in stages (I-III or IV) or for adenomyosis presence respectively highlighted a significant difference in terms of C1q expression, with higher C1q levels in the most severe group **(D)**, and in adenomyosis-positive EM patients **(E)**. C1q expression was evaluated by examining the collective mean of individual *C1QA*, *C1QB*, and *C1QC* genes. Data are expressed as box-plots (median, interquartile range). *p < 0.05 (unpaired two-tailed t-test).

To dissect further the role of C1q in EM, we isolated primary cells from patient-derived endometriotic lesions. After enzymatic digestion of the tissue, ECs were purified using magnetic beads coated with anti-CD31 (**Figure 4A**). As revealed by the IF (**Figure 4B**), the CD31^-^ cells consisted of a mixture of epithelial and stromal cells, called as endometriotic epithelial/stromal cells (EMCs), which positively stained for mucin-1/epithelial membrane antigen and vimentin, whereas the CD31^+^ endometriotic ECs (EECs) were absolutely positive for endothelial markers (i.e., CD31, VE-cadherin, vWF, and CD34; **Supplementary Figure 4**). Following the same protocol, we also isolated and characterized ECs from healthy ovary tissue (OVECs) as a control of acceptor tissue (**Supplementary Figure 4**).

Gene expression analysis using Real-Time quantitative PCR (RT-qPCR) revealed local expression of the three C1q genes (*C1QA*, *C1QB*, and *C1QC*) in all analyzed EM tissues, albeit with a notable degree of variability among samples. Conversely, neither EMCs nor EECs showed expression of C1q transcripts (**Figure 4C**). Peripheral blood mononuclear cells (PBMCs) were used as positive control and calibrator.

Interestingly, clustering EM patients based on disease severity (i.e., I-III stages and IV stage) revealed a significant difference in C1q expression between the two groups (I-III stages vs IV stage), with a higher level of C1q transcripts in the most severe group (**Figure 4D**). Moreover, significantly higher levels of C1q transcripts were found in patients presenting adenomyosis concomitant with EM (**Figure 4E**).

### C1q promotes angiogenesis in endothelial cells isolated from both endometriotic lesions and healthy ovary

**Figure 5:**
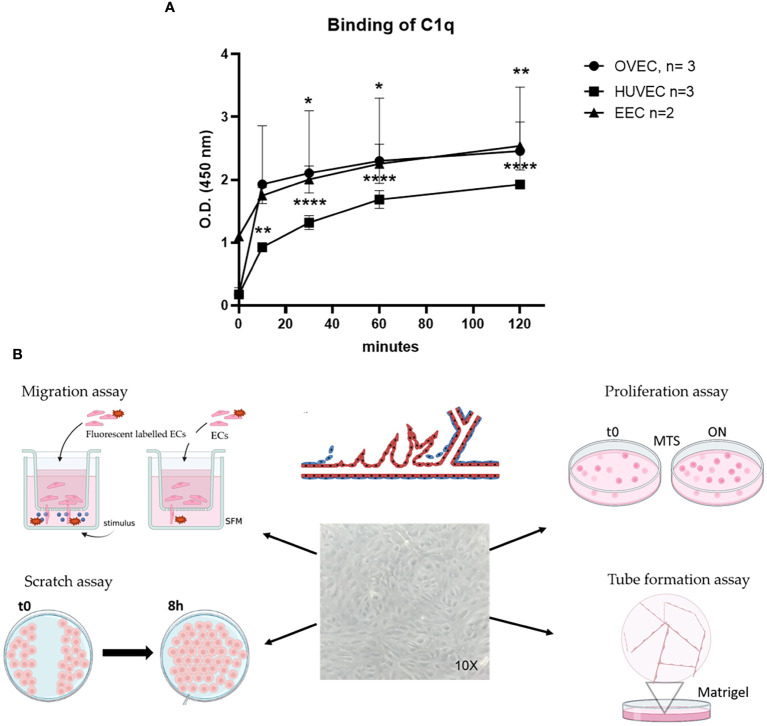
Binding of C1q to EECs, OVECs, or HUVECs. **(A)** Different endothelial cells [ECs; i.e., ECs isolated from healthy ovary (OVECs), *n* = 3; human umbilical vein ECs (HUVECs), *n* = 3; and endometriotic ECs (EECs)] grown to confluence on 96-well tissue culture plates were incubated with 10 µg/mL of purified C1q at different time points (0, 15, 30, 60, or 120 minutes) at room temperature. The binding of C1q was revealed by whole-cell ELISA assay. The data are presented as mean ± SD of three separate experiments. **(B)** Schematic representation of functional assays for the evaluation of C1q proangiogenic properties by migration, scratch, proliferation, and tube formation assays. Image created with BioRender.com, as an adaptation from Laschke et al. (33). *p<0.05; **p<0.01; ****p<0.0001.

To investigate the potential proangiogenic activity of C1q, we first assessed the ability of ECs to bind to C1q. We analyzed the behavior of EECs in comparison to OVECs, as a control of normal tissue, and to HUVECs, as a widely accepted model of ECs. The binding of C1q to ECs was evaluated by whole cell-ELISA assay, incubating live cells with C1q (10 µg/mL) for different time points (0, 15, 30, 60, or 120 min). As shown in **Figure 5A**, both EECs and OVECs reached the *plateau* after 10 min of incubation, whereas HUVECs did after 60 min. Furthermore, both these microvascular ECs were able to bind a higher amount of C1q compared to HUVECs.

**Figure 6:**
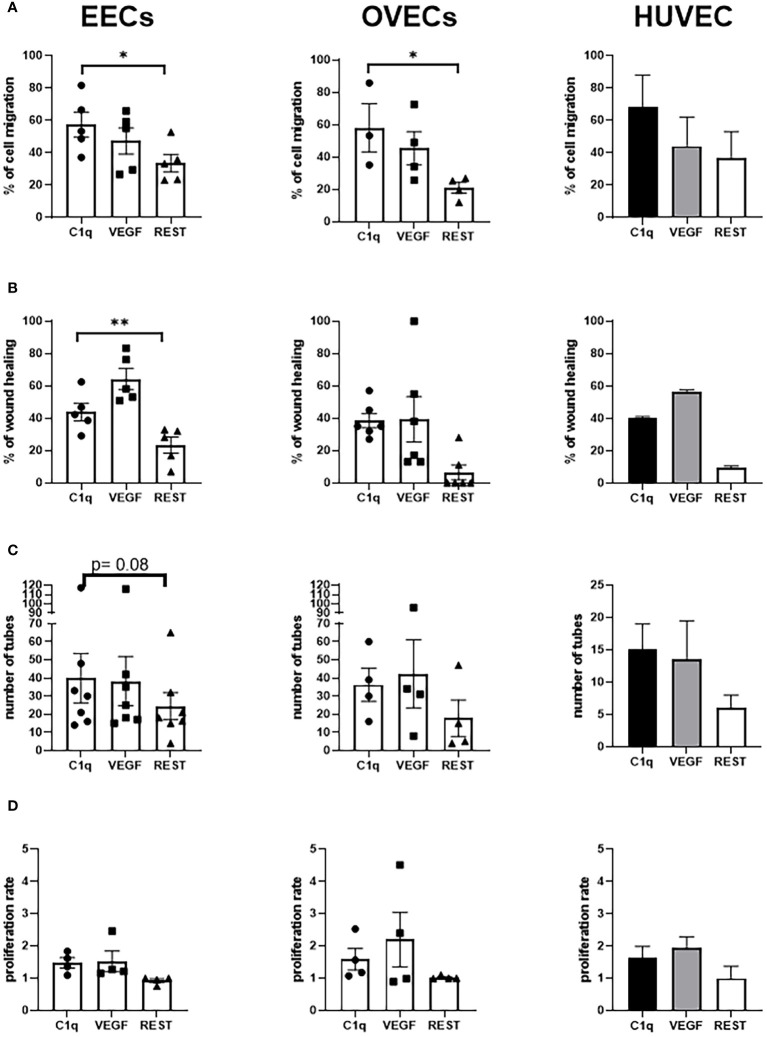
C1q promotes angiogenesis in EECs and OVECs. **(A)** Migration assays were performed in a trans-well system using endothelial cells (ECs) isolated from endometriotic cysts (EECs), healthy ovary (OVECs), or human umbilical vein (HUVECs). Cells were stained with FAST DiI™, seeded in FluoroBlok™ Inserts (1.5x10^5^ cells/insert), and the lower chamber was loaded with C1q (10 μg/mL) or VEGF (20 ng/mL), as chemoattractant stimuli. After 24h, fluorescence was read via INFINITE 200 Fluorescence Plate Reader. Data are expressed as mean ± standard deviation (SD). *p < 0.05; **p < 0.01. **(B)** Wound healing assays were performed using EECs, OVECs, and HUVECs. Cells (5x10^4^/well) were grown until 60–70% of confluence in a 24-well plate. After scratching the middle of endothelial monolayer, cells were stimulated with C1q (10 μg/mL) or VEGF (20 ng/mL). Images of the wound fields were captured after 18h, allowing calculation of percentage wound closure. Data are expressed as mean ± SD. *p < 0.05; **p < 0.01. **(C)** Tube formation assays were performed in EECs, OVECs, and HUVECs. Cells (5x10^4^) were seeded onto Matrigel^®^ in CultureSlides, and stimulated with C1q (10 μg/mL) or VEGF (20 ng/mL). After 18h, using TiEsseLab BDS 600 microscope, the capillary-like structures formed by ECs were manually counted, comparing the different conditions. Data are expressed as mean ± SD. *p < 0.05. **(D)** Proliferation assays were performed using EECs, OVECs, and HUVECs. Cells (7x10^3^/well) were seeded in a 96-well plate, and stimulated with C1q (10 μg/mL) or VEGF (20 ng/mL) for 24h. MTS was then added in each well, and cell proliferation was measured using PowerWave Select X Microplate Reader. Data are expressed as mean ± SD. REST, resting cells.

Next, to investigate a proangiogenic role of C1q in EM, we performed a series of functional assays using EECs or OVECs, involving migration, scratch, tube formation, and proliferation (**Figure 5B**). HUVECs were used as positive control. We also carried out the above-mentioned experiments in parallel using VEGF, a well-known proangiogenic factor. Migration and scratch assays revealed that C1q was able to significantly promote the motility of both EECs and OVECs, in a VEGF-comparable manner (**Figures 6A, B**). The results obtained with OVECs suggested that C1q could play a pivotal role also in physiological angiogenesis in the healthy ovary. These observations were further corroborated by tube formation assay (**Figure 6C**), where ECs were loaded onto a 3D matrix of Matrigel^®^ in the presence or absence (REST; resting cells) of the stimuli (C1q or VEGF). The capillary-like structures forming closed tubes were counted after 18h; EECs were found to form a higher number of tubes in all the experimental conditions compared to OVECs. Moreover, we analyzed the ability of C1q to enhance the proliferation using an MTS assay, which revealed that C1q indeed enhanced the cell proliferation rate (**Figure 6D**).

### C1q can induce endothelial cell motility via gC1qR

**Figure 7:**
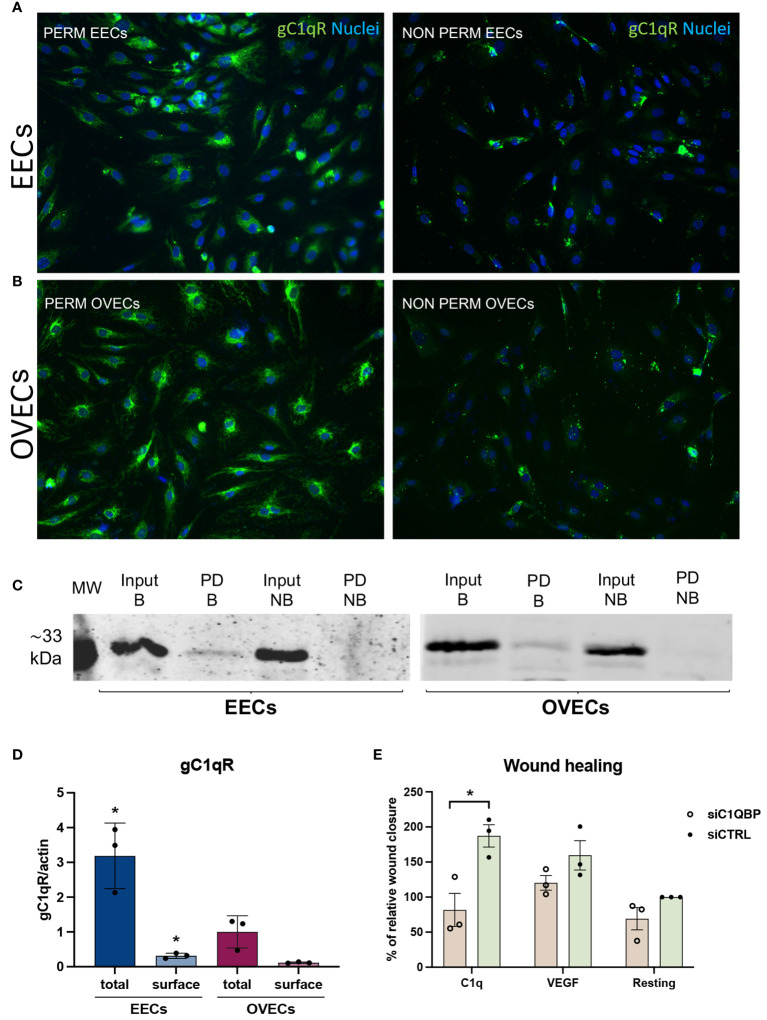
gC1qR is expressed in EECs and OVECs and can modulate C1q-induced proangiogenic behaviour. **(A, B)** Representative images displaying gC1qR positive staining (green) in EECs and OVECs, comparing permeabilized and not permeabilized cells. Cell nuclei were stained with DAPI (blue). **(C, D)** Surface biotinylation assay for the detection of gC1qR fraction present on the cell surface of EECs (*n* = 3) and OVECs (*n* = 3). Cells were treated with Sulfo-NHS-biotin reagent, biotinylated cell surface proteins were isolated upon binding to a Streptavidin-coated resin, and separated on a 10% SDS-PAGE. Membrane was then probed with anti-gC1qR antibody and anti-rabbit IRDye 800CW secondary antibody by Western blot analysis. Signal intensity was detected using an Odyssey CLx near-infrared scanner (LI-COR Biosciences, Lincoln, NE, USA). Image acquisition, processing, and data analysis were performed with Image Studio 5.2 (LI-COR Biosciences). β-actin was used to normalize the results. Data are expressed as mean ± standard deviation (SD). EECs displayed a higher amount of gC1qR compared to OVECs, considering both total protein and cell surface fraction; *p < 0.05. B, biotinylated; MW, molecular weights; NB, not biotinylated; PD, pull down. **(E)** Wound healing assay was performed using HUVECs (*n* = 3) after transfection with siC1QBP (gC1qR gene) or siCTRL for 48h. After scratching the middle of endothelial monolayer, cells were stimulated with C1q (10 μg/mL) or VEGF (20 ng/mL). Images of the wound fields were captured at 0 and 8h, percentage wound closure was calculated. The percentage of wound healing was considered as relative to siCTRL in resting conditions (100% of wound healing). Data are expressed as mean ± SD; *p < 0.05.

Given the effect of C1q on EECs, we aimed to elucidate the mechanisms underlying the proangiogenic capabilities of C1q by investigating the potential involvement of a putative C1q receptor. To this end, we characterized the expression of the receptor for the globular head of C1q (gC1qR), also known as hyaluronic acid-binding protein 1 (HABP1) or p32, in EECs and OVECs. First, IF was employed to detect gC1qR expression in both permeabilized and non-permeabilized ECs, as shown in **Figures 7A, B**. Remarkably, gC1qR exhibited elevated expression in permeabilized EECs and OVECs. Specifically, we observed a ubiquitous cytoplasmatic expression with a granular pattern, indicating potential co-localization with mitochondria. Interestingly, results from non-permeabilized cells also suggested the presence of gC1qR also at the cell surface, implicating its likely localization for C1q binding. To characterize the cell surface fraction of gC1qR, we carried out a cell surface biotinylation assay followed by SDS-PAGE and Western blot analysis (**Figure 7C**). The surface labeling with a biotinylated derivative confirmed gC1qR presence on the cell membrane of EECs as well as OVECs. Notably, we observed a higher amount of gC1qR in EECs considering both total protein and cell membrane fraction, compared to OVECs (**Figure 7D**).

To assess the functional involvement of gC1qR in C1q-dependent proangiogenic effects, we conducted short interfering RNA (siRNA) experiments in HUVECs as a model of EC behavior. Following 48h of silencing, HUVECs transfected with siC1QBP (gC1qR gene) or siCTRL were tested in wound healing assay using C1q or VEGF as stimulus. Silencing of gC1qR resulted in a significantly reduced response of HUVECs to C1q, as evidenced by a lower percentage of wound closure after scratching (**Figure 7E**). The findings confirm that proangiogenic effects of C1q is likely to be mediated through gC1qR. Conversely, no significant differences were observed after VEGF stimulation or under resting conditions.

## Discussion

Although an ever increasing body of scientific research has emerged on EM over recent years, the precise mechanisms governing its pathogenesis remain poorly understood, necessitating further investigation for the development of novel therapeutic strategies (34). The local and systemic immunological dysfunction is a well-established component in EM pathophysiology (35). In the current study, we enrolled a cohort of EM patients (*n* = 20), and we collected endometrial biopsies, a fragment of the ovarian cyst, and the peripheral blood. The latter was used to perform flow cytometric immunophenotyping, to analyze in particular the NK cell subset. In normal endometrium, CD56^bright^ uNK cells represent the major lymphocyte population (36). In EM, a decreased activity of peritoneal blood and peritoneal NK cells has consistently been reported, displaying reduced cytotoxicity and chemotaxis (37–41). It has been proposed that the abrogated NK cell function may be partially due to changes in the frequency of circulating CD56^+^ and/or CD16^+^ NK cells in EM patients, although a real consensus is yet to emerge (42). Interestingly, we observed a significantly higher percentage of circulating CD56^++/bright^ NK cells in EM patients compared to healthy women. This is consistent with an overall scenario of reduced NK cytotoxicity and defective immunosurveillance towards autologous cells.

A significantly impaired complement pathway in EM is widely acknowledged (15). In this study, we focused on the first recognition subcomponent of complement classical pathway, namely C1q, due to its involvement in angiogenic processes, which are fundamental for the maintenance of endometriotic implants (43). Sikora and colleagues have reported significantly higher levels of C1q, mannan-binding lectin (MBL), and C1-inhibitor in the peritoneal fluids of EM patients compared to control group (44). Recent multi-omics analysis also revealed an upregulation of *C1QA* expression in EM (45). Thus, we first examined the role of C1q in EM by gene expression analyses based on EndometDB. As previously observed for the complement component C3 (46), a higher expression of C1q genes was observed in EM tissues compared to uterine endometrium, in particular in OMA and peritoneal lesions. Interestingly, OMA manifested a correlation with the severity of the disease, suggesting an association between C1q expression and lesion development. Furthermore, we found a significantly increased expression of C1q in EM patients with concomitant adenomyosis. The main histologic feature of adenomyosis is the presence of endometrial glands and stroma within the myometrium (47), suggesting the key role played by angiogenesis in the pathogenesis of adenomyosis (48). The observation of higher levels of C1q transcripts in patients with more severe EM (IV stage) and those with concomitant adenomyosis is clinically relevant, and suggests a potential association between C1q expression and disease prognosis. However, due to the small sample size, this conclusion needs validation using larger cohorts.

Based on bioinformatics dataset, we further investigated C1q expression also at the protein level focusing exclusively on OMA. We observed the presence and the local synthesis of C1q in endometriotic lesions. IHC revealed a substantial presence of C1q in EM tissues, suggesting a likely activation of the complement classical pathway, only to be deterred by the very low intensity of C4d staining, as a byproduct of cascade activation. This observation, together with our previous findings concerning the role of C3 in EM (46), appears that the to suggest that complement activation in EM lesions mostly takes place via the alternative pathway. This is consistent with the involvement of the coagulation pathway in EM, which can directly activate C3 (*i.e.* by activating plasmin) (49). Complement activation at the EM lesion site can consistently contribute to local inflammation, chemotactic leukocyte infiltration, and damage to colonized healthy tissue.

We compared the C1q expression in EM lesions with the patients’ eutopic endometrium, as the likely tissue of origin of EM, and with healthy ovary, being the main engraftment site for ectopic endometrium. Surprisingly, eutopic endometrium was almost negative for C1q staining, while healthy ovary tissues showed a moderate positivity, indicating a potential role of C1q in physiological processes involving ovary. Based on the strong structural analogy between C1q and adipokines (belonging to the C1q/TNF superfamily) (18) and their close involvement in the regulation of sex hormone synthesis (50), it is possible that C1q may play a role in ovarian steroidogenesis as well as follicle maturation. Furthermore, Sang Wook Yoo and colleagues reported the presence of a range of complement components (i.e., C1qA, C1qB, C1qC, C1r, C1s, C2, C3, C4BP, C4A, C4B, C7, C8α, C8β, C9, Factor D, Factor H, FH-1, FH-5, Factor I, properdin, and MASP-1) in human follicular fluids, whereas C5 and C6 were notably absent (51). This evidence suggests that C1q could play a role in granulosa cell function during the follicular process and in oocyte maturation.

We tried to determine the local cellular origin of C1q in the EM lesions. As expected, double IF results highlighted a high grade of co-labeling with macrophages (CD68^+^ cells), but also unexpectedly revealed that ECs in their early stage of maturation (CD34^+^ cells) were able to synthesize C1q. Of note, since EM microenvironment is characterized by inflammation, leukocyte infiltration, and activation of coagulation, we cannot totally exclude a partial deposition of C1q derived from the blood circulation as well, in addition to local synthesis. Plasma is a possible source of C1q, interacting with the membrane of ECs. However, we think that the involvement of circulating C1q could be marginal because binding of plasma C1q to target cells usually leads to complement activation through the classical pathway, which apparently does not occur in EM, as evident from the low level of C4d deposition.

Accumulating evidence support the involvement of C1q in several physiological and pathological processes related to angiogenesis (16, 21, 23). Studies have highlighted the proangiogenic role of C1q in wound healing process (21, 52). Notably, C1q is localized in granulation tissue and, independently from complement activation, can stimulate permeability, proliferation, and migration of ECs. Additionally, C1q is involved in the angiogenic process during the post-stroke ischemia recovery (53). Thus, C1q could function as a proangiogenic factor, offering novel therapeutic possibilities (19).

Considering the previously established heterogeneity in ECs behavior across different tissue districts (25), we isolated primary ECs from endometriotic cysts in patient (EECs), and compared their response to C1q stimulation together with HUVECs and OVECs, the ECs isolated from healthy ovary. C1q promoted EC proliferation, migration, and tube formation.

Hayuningtyas et al. demonstrated that the collagen-like region of C1q induced the phosphorylation of DDR2 (Discoidin Domain Receptor 2), p38 kinase, and ERK (Extracellularly Regulated Kinase) 1/2 in a fibroblast cell line. Through the binding to DDR2, C1q enhanced fibroblast migration; DDR2-specific siRNA reduced C1q-mediated cell migration for wound healing (52). On the contrary, Bossi and colleagues showed that the main contribution to EC activation is attributable to the globular (gC1q) domain of C1q (21). In addition to achieving the same pro-angiogenic effects using only the globular portion of C1q, they demonstrated the neutralizing effect of a specific antibody against gC1qR, whereas an antibody against cC1qR (a putative receptor for the collagen region of C1q i.e. calreticulin) did not bring about a statistically significant inhibition. Therefore, we investigated the role of gC1qR in mediating the effect induced by C1q in the current study. We also identified gC1qR as a putative receptor involved in C1q-mediated angiogenic processes, being present on the cell surface of both EECs and OVECs. Interestingly, antibody neutralization of cell-surface gC1qR has been shown to inhibit angiogenesis, preventing cell migration and tube formation in HUVECs (54). Silencing of gC1qR gene via RNA interference could impair the ability of C1q to promote EC migration. The expression of cell-surface gC1qR at comparable levels may also justify the slight differences in terms of C1q binding or angiogenic response between EECs and OVECs. Moreover, the presence of C1q in the healthy ovary is likely attributable to its inherent proangiogenic role, which is a fundamental process in follicle maturation (55).

## Conclusions

C1q is abundantly present within endometriotic lesions and is expressed in normal ovary tissue as well, suggesting its potential role in physiological ovary angiogenesis. C1q in EM lesion microenvironment is expressed mainly by resident macrophages, although the ability to express C1q by ECs in an early phase of their differentiation still remains to be clarified. Our data demonstrate that C1q promotes angiogenesis of endometriotic and ovary ECs. C1q, supporting angiogenesis, appears as a central factor for the maintenance and growth of EM engraftment. Thus, this study opens up a new opportunity for identifying novel therapeutic targets for treating EM, possibly targeting C1q-gC1qR interaction that mediates angiogenesis in EM lesions.

## Data availability statement

The original contributions presented in the study are included in the article/**Supplementary Material**. Further inquiries can be directed to the corresponding authors.

## Ethics statement

The studies involving human subjects were approved by Regional Ethical Committee of FVG (CEUR), Udine, Italy (Prot. 0010143/P/GEN/ARCS 2019). The studies were conducted in accordance with the local legislation and institutional requirements. The patients provided their written informed consent to participate in this study.

## Author contributions

CA: Conceptualization, Formal analysis, Investigation, Methodology, Writing – original draft. MT: Data curation, Methodology, Software, Writing – original draft. GZ: Investigation, Methodology, Software, Writing – review & editing. AB: Data curation, Formal analysis, Investigation, Methodology, Software, Writing – original draft. SP: Methodology, Software, Validation, Writing – review & editing. MS: Methodology, Software, Writing – review & editing. LP: Investigation, Resources, Software, Validation, Writing – review & editing. FR: Methodology, Project administration, Software, Writing – review & editing. GL: Data curation, Software, Validation, Writing – review & editing. AM: Data curation, Formal analysis, Software, Writing – review & editing. AS: Data curation, Methodology, Validation, Writing – review & editing. BS: Data curation, Methodology, Software, Visualization, Writing – review & editing. EV: Data curation, Methodology, Resources, Writing – review & editing. GG: Data curation, Investigation, Methodology, Resources, Writing – review & editing. GR: Project administration, Resources, Supervision, Writing – review & editing. UK: Conceptualization, Formal analysis, Project administration, Resources, Writing – review & editing. RB: Funding acquisition, Investigation, Project administration, Supervision, Visualization, Writing – original draft.
