# Supplementary material for: Proangiogenic properties of complement protein C1q can contribute to endometriosis
Source: Front Immunol. 2024 Jun 25;15:1405597. doi: 10.3389/fimmu.2024.1405597 (PMC11231091; doi:10.3389/fimmu.2024.1405597)
Supplement: Supplementary file 1 [file DataSheet_1.docx]

Supplementary Material to

**Proangiogenic properties of the complement protein C1q can contribute to endometriosis**

Chiara Agostinis^1^, Miriam Toffoli^2^, Gabriella Zito^1^, Andrea Balduit^1*^, Silvia Pegoraro^1^, Mariagiulia Spazzapan^3^, Lorella Pascolo^1^, Federico Romano^1^, Giovanni Di Lorenzo^1^, Alessandro Mangogna^1^, Aurora Santin^2^, Beatrice Spedicati^1,2^, Erica Valencic^1^, Giorgia Girotto^1,2^, Giuseppe Ricci^1,2^, Uday Kishore^4,5*^, Roberta Bulla^3^

^1^ Institute for Maternal and Child Health, IRCCS Burlo Garofolo, Trieste, Italy;

^2^ Department of Medical, Surgical and Health Science, University of Trieste, Trieste, Italy;

^3^ Department of Life Sciences, University of Trieste, Trieste, Italy;

^4^ Department of Veterinary Medicine, United Arab Emirates. University, Al Ain, U.A.E.;

^5^ Zayed Centre for Health Sciences, United Arab Emirates (UAE) University, Al Ain, U.E.A.

**Supplementary Tables**

**Supplementary Table 1.** **Percentage values of peripheral blood mononuclear cell immunophenotyping in endometriosis (EM) patients and healthy controls (CTRL).**

|  | **% tot Leucocytes** | **% CD45^++^ Lymphocytes** | | | |
| --- | --- | --- | --- | --- | --- |
|  | **CD45^++^** | **CD3^+^** | **NK (CD3^-^ CD56^+^)** | **NK CD56^+/dim^** | **NK CD56^++/bright^** |
| **CTRL** | **29.1 (3.1)** | **67.0 (7.0)** | **9.2 (3.5)** | **7.8 (7.8)** | **0.6 (0.6)** |
| **EM** | **27.9 (6.4)** | **69.4 (8.1)** | **11.1 (6.0)** | **9.6 (5.3)** | **1.0 (0.6)** |

Data are expressed as mean (SD) of EM (*n* = 17) compared to CTRL women (*n* = 8). NK, natural killer.

**Supplementary Figures**

**Supplementary Figure 1**

**
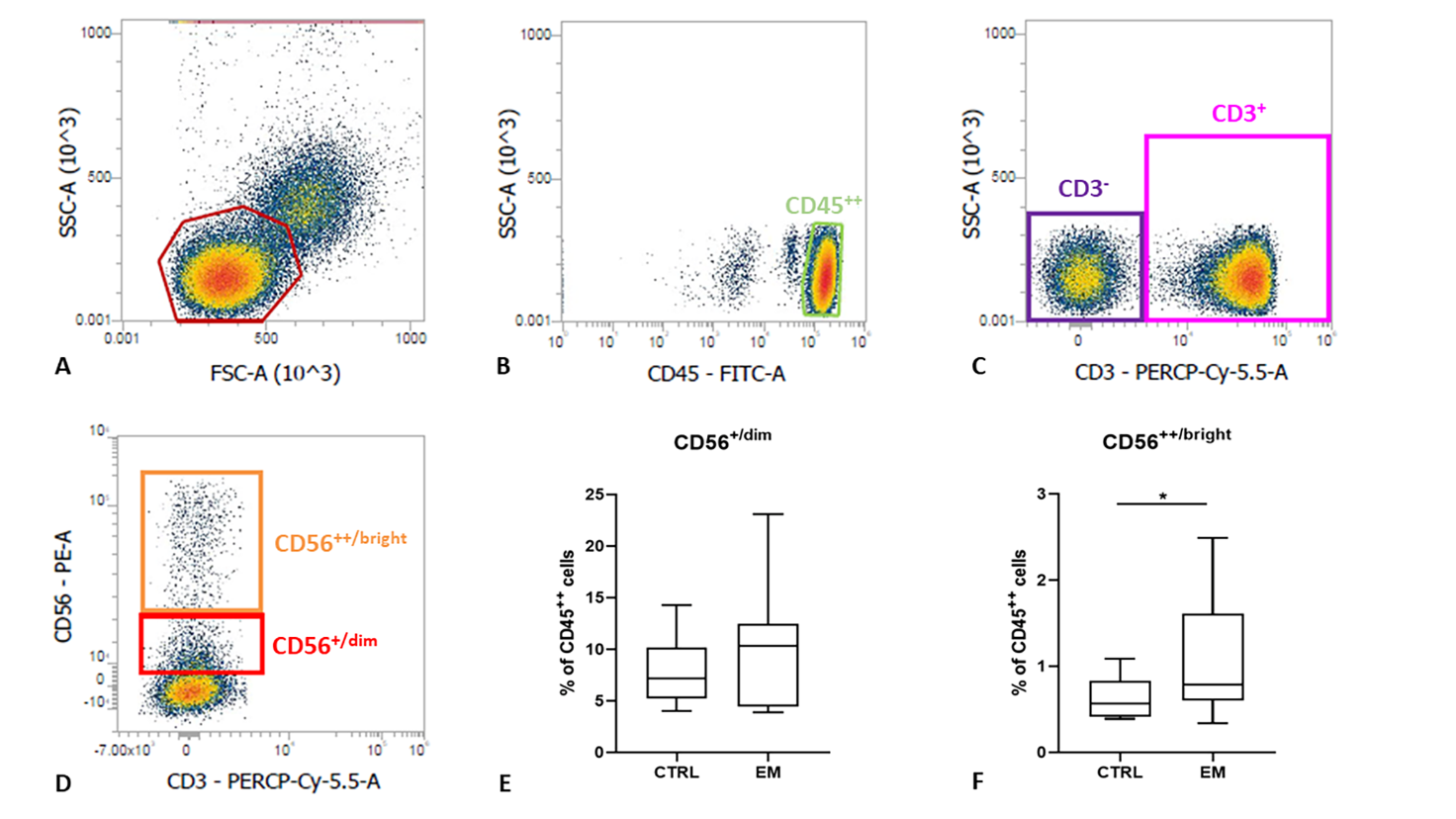
Supplementary Figure 1.** **Immunophenotyping of peripheral blood mononuclear cells in EM patients and healthy controls**. Representative density plots displaying the peripheral blood mononuclear cells (PBMCs)’ gating strategy used to identify and characterize natural killer (NK) cells. Lymphocytes were identified based on their forward scatter (FCS) and side scatter (SSC) characteristics (**A**). Among CD45^++^lymphocytes (**B**), only CD3^-^ cells were selected (**C**), and NK cells were further divided into CD56^++/bright^ and CD56^+/dim^ (**D**). A significantly increased number of CD56^++/bright^ cells was detected in EM patients (*n* = 17) compared to controls (*n* = 8), whilst no significant differences were observed in CD56^+/dim^ NK cells (**E**-**F**). **p* < 0.05.

**Supplementary Figure 2**

**
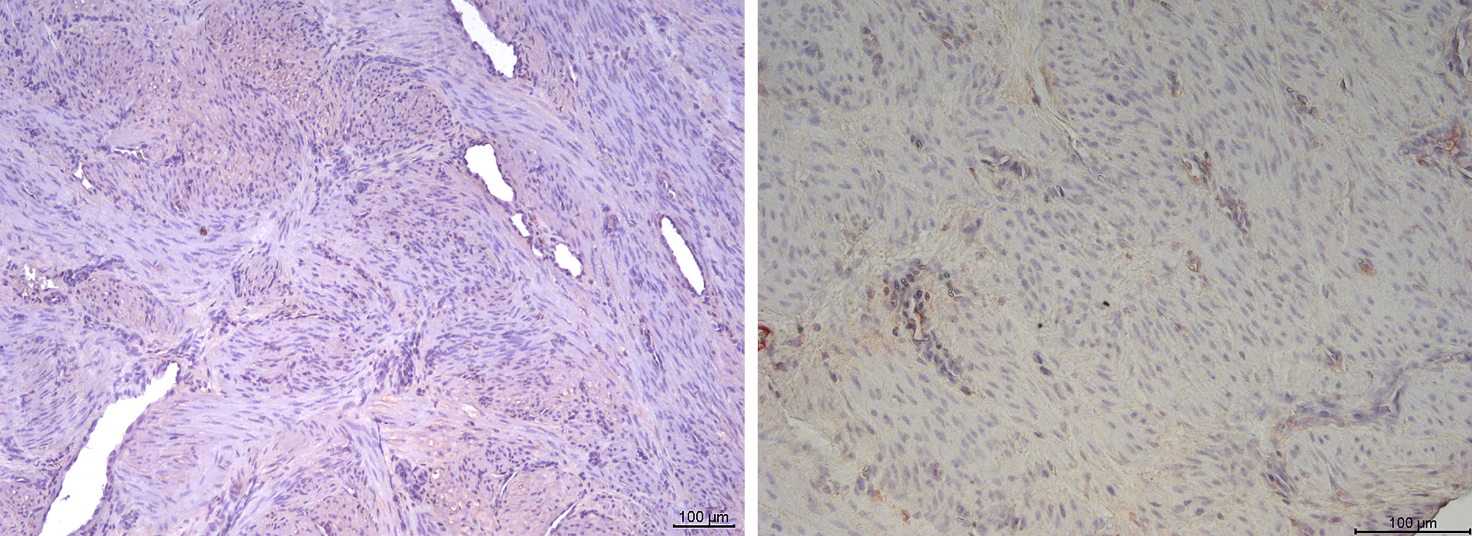
**

Representative microphotographs showing the presence of C1q in normal endometrium. AEC (red) chromogen was used to visualize the binding of rabbit anti-human C1q antibody. Nuclei were counterstained in blue with Harris Hematoxylin. Magnification, 10x (left panel), 20x (right panel). Scale bars, 100 µm.

**Supplementary Figure 3**


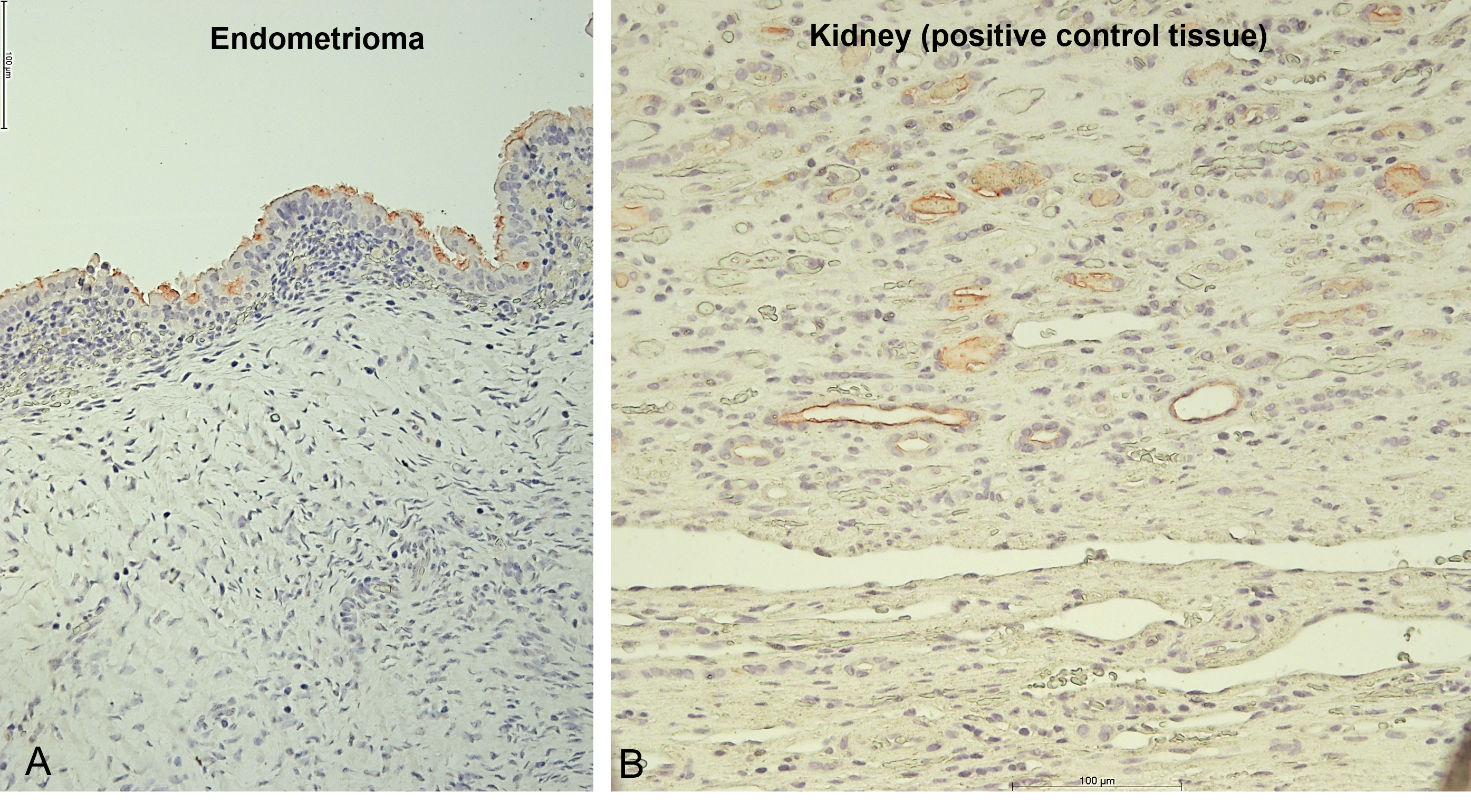
**Supplementary Figure 3.** Microphotographs of CD133 expression in endometrioma (**A**) or kidney (**B**) as positive control tissue. AEC (red) chromogen was used to visualize the binding of secondary antibodies. Nuclei were counterstained in blue with Harris Hematoxylin. Magnification, 10x. Scale bars, 100 µm.

**Supplementary Figure 4**
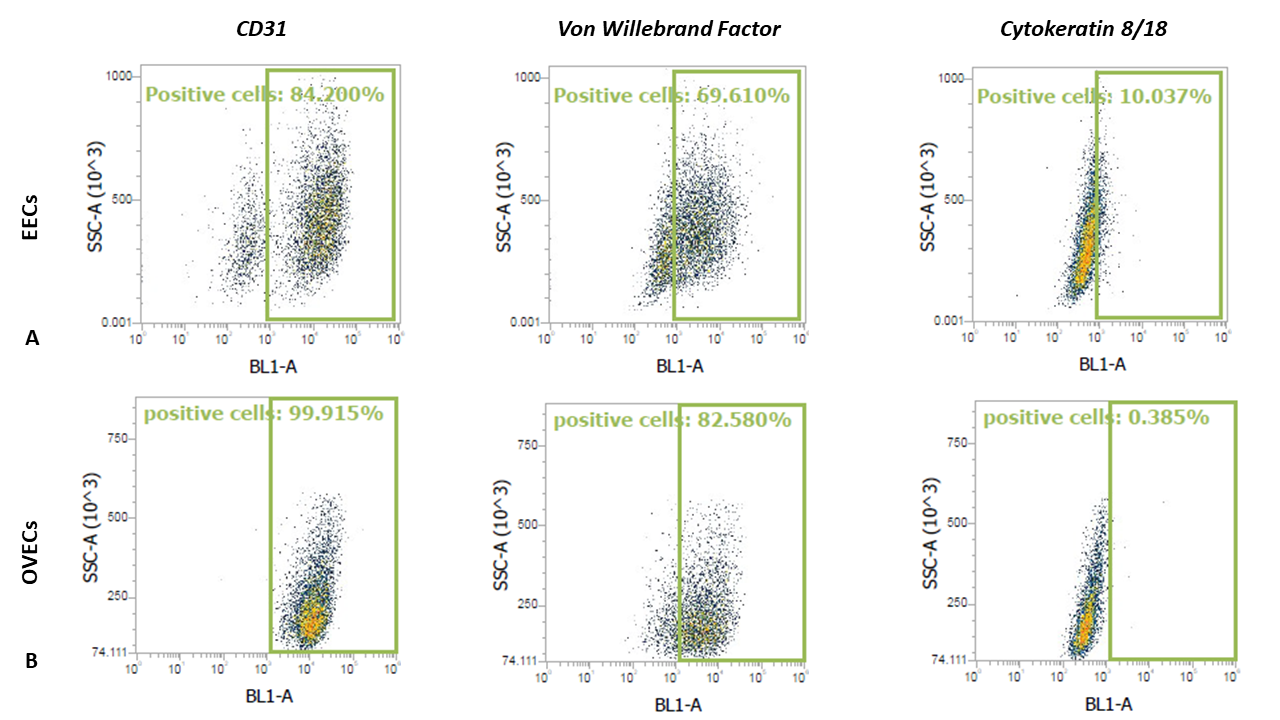


**Supplementary Figure 4.** Characterization of endothelial cells isolated from EM ovary cysts (EECs) and from healthy ovary (OVECs) by cytofluorimetric analysis. The cells were labelled with two major endothelial markers, CD31 and von Willebrand Factor (vWF), and with an epithelial cell marker, cytokeratin 8/18, to assess the purity of the isolated endothelial population. Strong positivity for CD31 and VWF was observed, particularly in OVECs, while in both cases, cytokeratin expression was very low, confirming the purity of the populations obtained with the isolation method used in this study. Fluorescence was acquired using the Attune NxT Flow Cytometer (ThermoFisher) equipped with a Blue Laser (488nm) and analyzed with the Attune Cytometric Software v5.3.0.
